# Parameters influencing the size of chitosan-TPP nano- and microparticles

**DOI:** 10.1038/s41598-018-23064-4

**Published:** 2018-03-16

**Authors:** Sruthi Sreekumar, Francisco M. Goycoolea, Bruno M. Moerschbacher, Gustavo R. Rivera-Rodriguez

**Affiliations:** 1 Institute of Plant Biology and Biotechnology (IBBP), Westfälische Wilhelms-Universität Münster, Schlossplatz 8, 48143, Münster, Germany; 2 Present Address: School of Food Science and Nutrition, University of Leeds, LS2 9JT, Leeds, UK

## Abstract

Chitosan nanoparticles, produced by ionic gelation, are among the most intensely studied nanosystems for drug delivery. However, a lack of inter-laboratory reproducibility and a poor physicochemical understanding of the process of particle formation have been slowing their potential market applications. To address these shortcomings, the current study presents a systematic analysis of the main polymer factors affecting the nanoparticle formation driven by an initial screening using systematic statistical Design of Experiments (DoE). In summary, we found that for a given chitosan to TPP molar ratio, the average hydrodynamic diameter of the particles formed is strongly dependent on the initial chitosan concentration. The degree of acetylation of the chitosan was found to be the second most important factor involved in the system’s ability to form particles. Interestingly, viscosimetry studies indicated that the particle formation and the average hydrodynamic diameter of the particles formed were highly dependent on the presence or absence of salts in the medium. In conclusion, we found that by controlling two simple factors of the polymer solution, namely its initial concentration and its solvent environment, it is feasible to control in a reproducible manner the production and characteristics of chitosan particles ranging in size from nano- to micrometres.

## Introduction

The best-studied chitosan-based nanocomposites are probably nanoparticles produced by ionotropic gelation^1^, utilizing the sol-gel transition of chitosan polymers in the presence of a poly-anionic crosslinking agent, typically sodium tripolyphosphate (TPP). The mild and aqueous processing conditions, the non-toxic reagents, and the very low energy requirements make this production route the best suited for any biological application, such as oral drug delivery^2^, protein formulation^3^, and gene therapy^4^, to mention a few. Despite this well-established protocol for the preparation of chitosan nanoparticles, the influence of different processing factors on the size of the particles is not yet well known. Even though many researchers have tried to optimize the processing parameters in order to control the ionic gelation process in a robust way and, thus, the size of the particles obtained^2,5–8^, a systematic study is still missing.

Chitosans are a family of functional biopolymers that can be obtained by partial de-*N*-acetylation of the highly abundant, naturally occurring polymer chitin^9^. Chitosans are composed of β-1,4-linked glucosamine and *N*-acetylglucosamine residues. Both the degree of acetylation (DA) and the degree of polymerisation (DP) of chitosans are crucial factors known to govern the structural and functional properties of this family of polymers^10^ and their resulting engineered materials^11^. Commercially available chitosans vary in their DA, typically between 5 and 20%, and in their DP or molecular weight, typically ranging between 10 and 500 kDa.

Even though structurally, chitosans are relatively low complexity polysaccharides, their solution behavior is rather complex, and their biological activities are manifold. Chitosans are gel- and film-forming polymers, they can bind metal ions and organic compounds in water filtration, they are biodegradable and biocompatible, they are mucoadhesive and have anti-microbial, plant strengthening, and scar-free wound healing activities, among others. One reason for these diverse functionalities is the presence of multiple free amino groups on the glucosamine residues generated when chitosan is produced from fully acetylated chitin by partial de-*N*-acetylation. These glucosamine residues carry positive charges at slightly acidic pH values, making chitosans poly-cationic biopolymers which can easily interact with poly-anionic molecules such as many proteins, DNA, or phospholipids. Also, the amino groups lend themselves for easy functionalization, further broadening the potential applications of chitosans. Chitosans, thus, are excellent candidates for the development of applications in the fields of pharmaceutics, food, agriculture, and bio-medicine^5^. Biomedical applications include the use of chitosans as a component of more complex materials, such as scaffolds or nanocomposites^12–15^. However, the main drawback when using chitosan-based nanosystems for biomedical applications is the poor understanding of their production protocols leading to poor reproducibility in the characteristics of the particles formed. This is because most studies focused on the particle properties as influenced by different chitosan-TPP mass ratios^7,16,17^ but did not take into account other experimental factors such as the DA of the chitosan used which can be expected to be highly significant in a process that depends on the NH_3_^+^ groups for interaction, like in the case of ionic gelation.

Therefore, the purpose of this study was to gain fundamental knowledge on the polymer factors and their interaction parameters that drive the formation of chitosan nanoparticles and influence their properties. The size of the particles obtained was varied by tuning the key parameters DA and DP, the NH_2_/PO_4_ molar ratio, and the concentration of the chitosan solution, using experimental design models. Unlike conventional approaches using a large number of independent experiments, this method enabled us to simultaneously test an array of factors ^19,19^ and to thus screen the processing conditions.

## Materials and Methods

### Materials

Fully deacetylated chitosan polymer was kindly provided by Gillet Chitosan (France). All other reagents used were of analytical grade and supplied by Sigma-Aldrich (Germany). Ultrapure Milli-Q water was used throughout.

### Methods

In this work, a series of chitosan/TPP nanoparticles was prepared by modifying some parameters in the polymer characteristics, namely degree of acetylation (DA), degree of polymerisation (DP), and the ratio of polymer to crosslinker. A detailed analysis of the influence of these parameters was carried out by using statistical approaches to identify critical factors and cross-interactions among the factors studied. Chitosan nanoparticles in this study were prepared by the well-known ionotropic gelation technique, using a series of chitosans prepared by partial chemical re-*N*-acetylation, and characterised using standard procedures.

#### Production of Chitosans

Routinely, raw chitosans with a DA around 0% were purified before depolymerization and re-*N*-acetylation as follows: chitosan was dissolved in 0.1 M acetic acid and filtered through a borosilicate filter series, (Robu Glasfilter-Geräte GmbH) (porosity ranging from 1 to 4). Finally, chitosan was precipitated using 23% (w/v) ammonia followed by rinsing with deionized water until neutral pH was reached. Finally, the pellet was freeze-dried overnight. The final product was weighed, characterized for DA and DP as described below, and stored under dry conditions (sample numbers 1 and 8 in Table 1).

**Table 1:** Structural characteristics of the chitosans used.

sample number	DA^a^ (%)	M_w_^b^ (kDa)	M_n_^b^ (kDa)	DP	I_p_^b^
1	1.5	210	116	1300	1.80
2	11	223	91	1300	2.45
3	18	166	99	1000	1.67
4	20	160	n.d	1000	n.d
5	35	227	125	1200	1.82
6	50	221	120	1200	1.83
7	60	243	152	1300	1.59
8	1.6	436	315	2700	1.38
9	35	450	182	2600	1.87
10	35	280	n.d	1600	n.d
11	35	125	58.8	700	1.80

^a^Degree of acetylation (DA) as determined by ^1^H NMR.

^b^Parameters determined by HPSEC-MALLS-RI: weight-average molecular weight (M_w_); number-average molecular weight (M_n_); polydispersity (I_p_ = M_w_/M_n_). n.d stands for values that were not determined.

Chitosans of different molecular weights were prepared by partial oxidative depolymerisation of chitosan 8 by using NaNO_2_ at room temperature^20^. Briefly, a chitosan solution of 10 mg/mL in acetic acid was mixed with 0.1 M NaNO_2_. The reaction was stopped at different time points, depending on the desired DP, and the chitosans produced were precipitated, washed, freeze-dried, and. characterized as above (samples 9–11).

Chitosans of different DA were prepared by partial re-*N*-acetylation using acetic anhydride as previously described^21^ in different molar ratios to reach the desired DAs. Briefly, chitosan 1 was solubilized in water by adding acetic acid in stoichiometric ratio. Once chitosan was dissolved, one volume of 1,2–propanediol was added to the chitosan solution in order to reduce the isoelectric constant of the medium and to help chitosan chains to adopt an open conformation. Then, acetic anhydride was added in the required molar amount to reach the target DA. After 12 h, chitosan was precipitated, washed, freeze-dried, and characterized as above (samples 2–7).

#### Characterization of Chitosans

The DA of the different chitosan samples was determined using ^1^H-NMR spectroscopy. Briefly, chitosan samples were dissolved in D_2_O with the aid of DCl (at pD 3–4) as described previously^22^. Spectra were recorded on a Bruker-Spectrospin AM 300 spectrometer (300 MHz). Approximately 200–250 scans were acquired.

The weight-average molecular weight (M_w_) and the number-average molecular weight (M_n_) of the different chitosan samples were measured by size exclusion chromatography using a SEC system from PSS (Polymer Standards Service GmbH, Germany), and the polydispersity was calculated from these data (I_P_ = M_w_/M_n_). The system consisted of an Agilent 1200 HPLC system with isocratic pump and a series of Novema columns protected with a pre-guard column. The order of the columns was set up as 30 Å, 3000 Å, 3000 Å. (I.D. 8 mm). To determine the concentration, the system used an online refractive index detector (Agilent 1200 RID) by setting the dn/dc values determined independently for each chitosan using interferometry (NFT ScanRef, Germany). Finally, for the size determination, a multi-angle-laser-light-scattering instrument (PSS SLD 7000 MALLS, Brookhaven Instruments), equipped with a 5 mW He/Ne laser operating at λ = 632.8 nm was used. The degassed mobile phase consisted of 0.2 M ammonium acetate/0.15 M acetic acid buffer with a pH of 4.5 at a flow rate of 0.7 mL/min at 35 °C. Data were collected and evaluated using the software WinGPC 7.0.1 (PSS Polymer, Germany). Structural characteristics of all chitosans used are summarized in Table 1.

The dynamic viscosity of chitosan aqueous solutions, solubilized with 5% stoichiometric excess of acetic acid, was measured using an AMVn automated rolling ball microviscosimeter (Anton Paar, Ostfildern, Germany) with programmable tube angle that works on the principle of the rolling ball time, the time taken by a steel ball to roll through the mixture inside a calibrated 1.6-mm diameter capillary. All measurements were performed at 25 °C, and for each sample the calculated dynamic viscosity was an average of four runs measured at an angle of 30°. The viscosity value was expressed as the relative viscosity (η_rel_), with respect to solvent, 5% stoichiometric excess of acetic acid, or as the specific viscosity (η_sp_ = η_rel_ − 1). Intrinsic viscosity ([η]) is the measure of dynamic viscosity of the solution at different concentrations and was calculated by joint extrapolation to “zero concentration” of the Huggins (Eq. (1)), Kraemer (Eq. (2)) and Single point (Eq. (3)) relationships^23^, respectively given as:

1 $$\frac{{\eta }_{sp}}{c}=[\eta ]+k\text{'}{[\eta ]}^{2}c$$

2 $$\frac{ln{\eta }_{rel}}{c}=[\eta ]+k\text{'}\text{'}{[\eta ]}^{2}c$$

3 $$[\eta ]=\frac{{\{2\times ({\eta }_{sp}-ln{\eta }_{rel})\}}^{1/2}}{c}$$

where c is concentration, and *k*′ and *k*″ are the Huggins and Kraemer constants, respectively.

#### Preparation of Chitosan Nanoparticles

Chitosan nanoparticles were prepared by ionic gelation with TPP crosslinking as described previously^1^, with slight modifications. Briefly, chitosan was dispersed in water and solubilized with a 5% molar excess of acetic acid with respect to the amino groups in the chitosan used. A stock solution of TPP in double distilled water (5 mg/mL) was prepared. Chitosan and TPP stock solutions were filtered using 0.45 µm and 0.22 µm pore size membrane filters, respectively. Chitosan nanoparticles were spontaneously obtained when TPP solution was added dropwise into the chitosan solution under magnetic stirring (750 rpm) at room temperature. Unless specified otherwise, particle synthesis was carried out with water as the solvent.

The size of the particles formed was determined using dynamic light scattering (DLS) with backscattering at 173° using the method of cumulants with a Zetasizer Nano ZS^TM^ (Malvern Instruments, UK). Different concentrations of chitosan and TPP solutions were mixed in order to produce different NH_2_/PO_4_ molar ratios as indicated below, keeping the volume ratio (3:1), stirring speed (750 rpm), and temperature (25 °C) constant, to determine the optimal NH_2_/PO_4_ ratio for each chitosan.

Using the optimal NH_2_/PO_4_ ratio for each chitosan, different concentrations of chitosan (and, consequently, TPP) were used to produce particles, with the same volume ratio, stirring speed, and temperature as given above. Chitosan concentrations ranging from 0.1 mg/mL to 5 mg/mL were studied.

#### Design of Experiments

A full factorial design was used to study the main factors influencing the production of chitosan nanoparticles. The studied factors, selected accordingly to previous information^24^, were the NH_2_/PO_4_ molar ratio, the chitosan concentration, and the degree of acetylation. Each factor was studied at three different levels, namely NH_2_/PO_4_ molar ratio at 0.5, 1, and 1.5, the chitosan concentration at 0.5 mg/mL, 2.75 mg/mL, and 5 mg/mL, and the DA at 20%, 35%, and 50%. Consequently, 3³ = 27 experiments were performed, each in triplicate.

The factorial design was created using Statgraphics XV (Statpoint Technologies), the variables were codified, in unitless values, to weigh the standardized influence of each of the factors. The resulting design is summarized in Table 2. We added two extra values of DA outside of the design limits in order to corroborate the validity of our design of space, namely DA 10% and DA 60%.

**Table 2:** Design of Experiments with codified and non-codified factor levels for the full factorial design.

factor	codified level			non-codified level		
	low	medium	high	low	medium	high
chitosan/TPP molar ratio	−1.0	0	1.0	0.5	1	1.5
chitosan concentration (mg/mL)	−1.0	0	1.0	0.5	2.75	5
DA of chitosan (%)	−1.0	0	1.0	20	35	50

For the definition of a model, and to identify the factors most strongly influencing particle production, the response results were fitted using different mathematical models, namely a linear regression, a full quadratic model, a cubic model, or a special cubic model, as a function of the studied factors and their interactions. In all cases, mathematical transformations to the factors were carried out in order to find the best explaining model. The best fitting model was selected by simply evaluating the R-square and the distribution of the residuals.

Possible effects of process factors other than those described in the factorial design should be considered as part of the calculated error for the experimental model. The experimental error was assumed to be random and, therefore, the error can be considered estimable through replicate studies at the center of the design. Three experiments were conducted at the design center to estimate the magnitude of the error in the experimental analysis. The experimental runs were carried out in a random manner to avoid any systematic bias in the results. Moreover, the produced residuals were analyzed to verify the randomness of the measurements.

An analysis of variance (ANOVA) of the experimental response was conducted to evaluate the best explanatory model of the full factorial design response surface model. The F-values and the p-values were used to define the order of the model (linear, square, cubic, or special cubic). The results of this estimation are reported by the Pareto charts. Furthermore, an analysis of residuals was used to verify the model. For a well-predicted model, the residuals are expected to follow a normal distribution^25^ and to have a R-square value above 70%.

#### Transmission electron microscopy

Particles were visualized using transmission electron microscopy (TEM). To this end, 10 µl of sample was diluted in water (1:100 v/v), then 10 μl of the diluted sample was mixed with 10 μl of 1% (w/v) uranyl acetate for 30 s. Then, 10 µl of the sample was placed onto a copper grid covered with a Formvar® film (200 mesh) for 30 s. Excess liquid was removed using a filter paper and the grids were dried in a desiccator for at least 24 h. Imaging was performed using Philips TEM CM10 (Eindhoven, Netherlands) fitted with a bottom mounted camera TVIPS TEMCam F416.

## Results and Discussion

Using a range of well-defined chitosans (see Table 1), different particulate formulations were prepared by changing the preparation conditions following a full factorial design. Factors that influence the size of the chitosan particles obtained were evaluated using factorial plots: main effect, interaction effect, Pareto chart plot, normal probability plots, and surface plot. ANOVA was used to check the significance of the effect on particle size. The main effects of factors and interactions were also observed in the Pareto chart plot.

Within the chosen design space, it was possible to produce chitosan particles ranging in average hydrodynamic diameter from 200 to 2500 nm. For a better understanding and for a more accurate analysis, the results were grouped according to the NH_2_/PO_4_ molar ratio used in the preparation of the particles, as this was previously described to be a significant factor in chitosan:TPP nanoparticle formation^24,26^. We found some conditions in which it was not possible to form particles, due to precipitation or unstable particle formation upon addition of TPP. This was the case at NH_2_/PO_4_ molar ratio of 0.5, independent of the chitosan’s DA. This observation was related to the excess anionic charges in comparison to the cationic charges available in the system, thus indicating that particle formation requires higher NH_2_/PO_4_ molar ratios.

**Figure 1:**
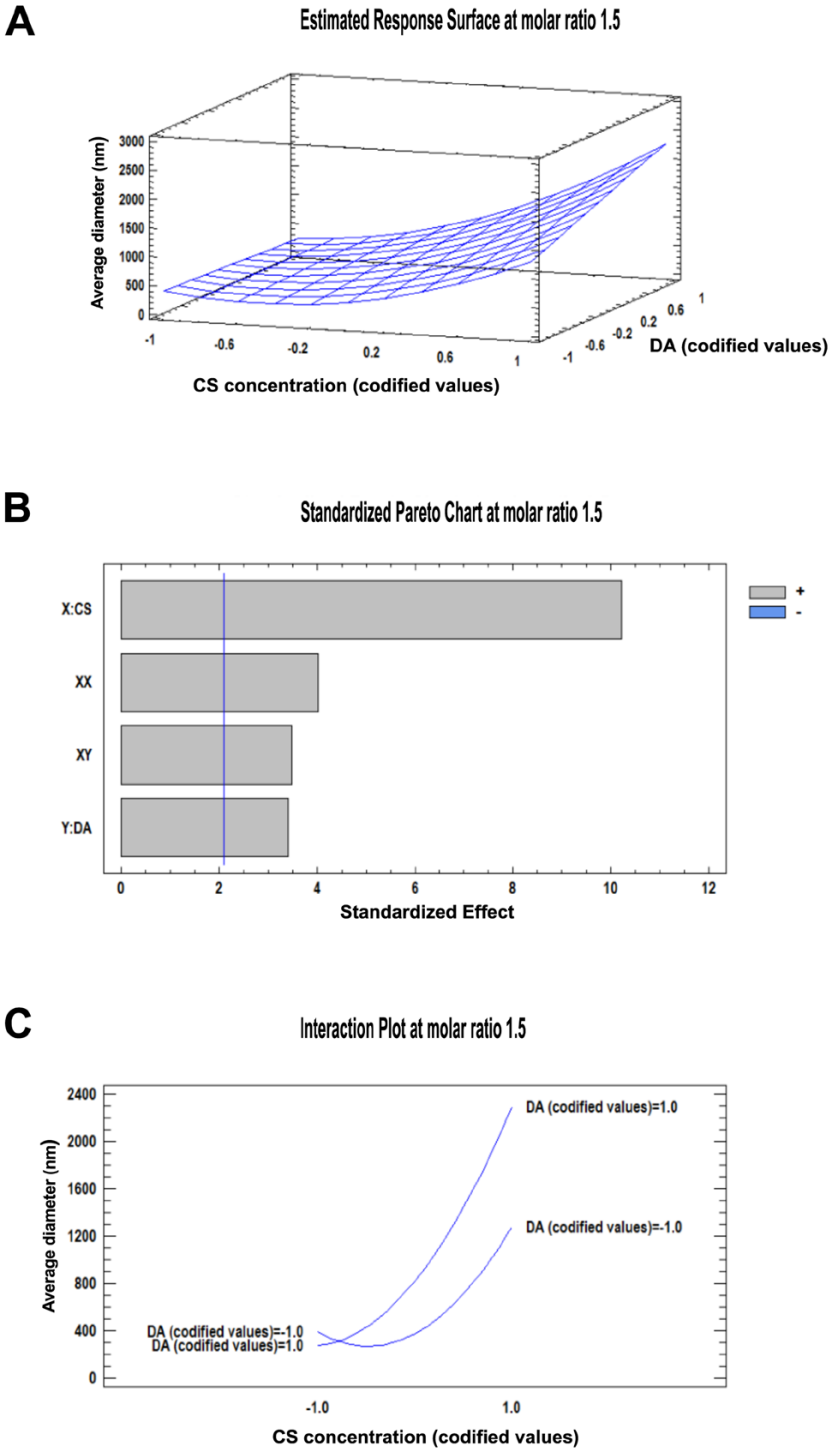
Surface response for influence on average hydrodynamic diameter of particles with a molar ratio of 1.5. (**A**) Response surface graph of the influence of chitosan concentration and DA on average hydrodynamic diameter; (**B**) Standardized Pareto chart estimating the effect of chitosan concentration, DA, and their interaction on the average hydrodynamic diameter of the particles. The blue vertical line represents the 0.05 critical value for ANOVA. All bars extending to the right of this line indicate that the effects are statistically significant at 5% significance level; X: chitosan concentration, Y: degree of acetylation, XX: interaction effect of chitosan concentration, XY: interaction effect of chitosan concentration and degree of acetylation. (**C**) Interaction plot of the studied factors on the average hydrodynamic diameter of the particles.

**Figure 2:**
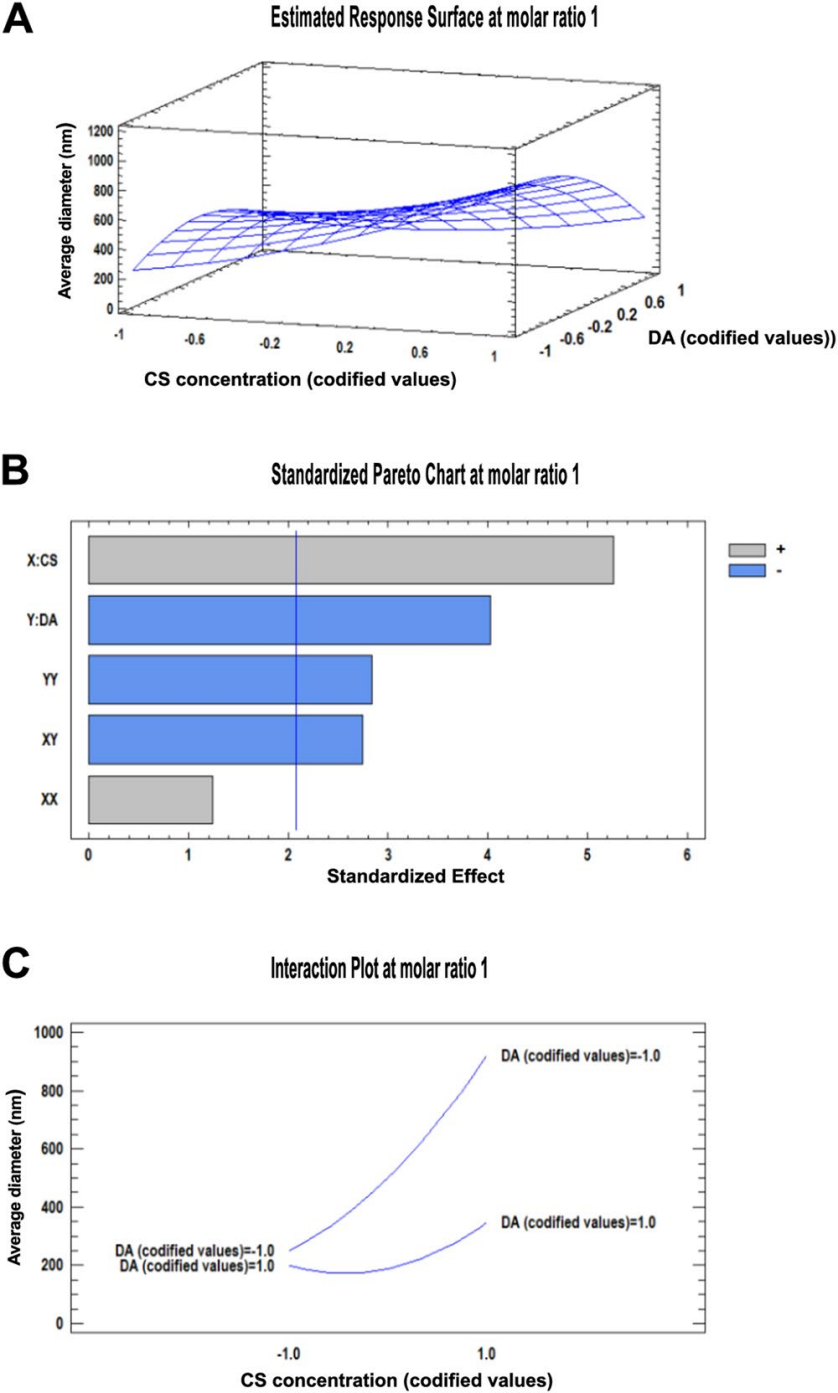
Surface response for influence on average hydrodynamic diameter of particles with a molar ratio of 1. (**A**) Response surface graph of the influence of chitosan concentration and DA on average hydrodynamic diameter; (**B**) Standardized Pareto chart estimating the effect of chitosan concentration, DA, and their interaction on the average hydrodynamic diameter of the particles. The blue vertical line represents the 0.05 critical value for ANOVA. All bars extending to the right of this line indicate that the effects are statistically significant at 5% significance level; X: chitosan concentration, Y: degree of acetylation, XX: interaction effect of chitosan concentration, XY: interaction effect of chitosan concentration and degree of acetylation, YY: interaction effect of degree of acetylation. (**C**) Interaction plot of the studied factors on the average hydrodynamic diameter of the particles.

The fitted response surface graphs at NH_2_/PO_4_ molar ratios of 1.5 and 1 are shown in Figs 1A and 2A, respectively. They represent the influence of both the concentration and the DA of the chitosan used on the average hydrodynamic diameter of the particles. In both cases, a good correlation to the quadratic model was observed in the coefficient analysis.

Figures 1B and 2B represent the standardized Pareto charts. They contain a bar for each effect, sorted from most significant to least significant. The length of each bar is proportional to the standardized effect. A vertical line is drawn at the location of the 0.05 critical value for the statistical test. Any bars that extend to the right of that line indicate effects that are statistically significant at the 5% significance level^27^. At a NH_2_/PO_4_ molar ratio of 1.5 (Fig. 1B), every variable studied here had a positive influence on the response variable, meaning that an increase in those produces an increase in the average hydrodynamic diameter of particles. It is evident that chitosan concentration had the strongest influence over the increment of particle size. We found that particles prepared from chitosans with DA 20% and 50% showed a broad size range from nano- to micrometers in hydrodynamic diameter when the chitosan concentration was increased from 0.5 mg/mL to 5 mg/mL. In contrast, for a NH_2_/PO_4_ molar ratio of 1 (Fig. 2B), only DA 35% displayed a similar relationship of the average hydrodynamic diameter of the particles formed to chitosan concentration. Unlike at the molar ratio of 1.5, at molar ratio 1 some factors also had a negative influence on the particle diameter (represented in blue bars in Fig. 2B). However, as seen before, the strongest factor influencing the average hydrodynamic diameter of the particles was chitosan concentration. It should be noted that other factors not varied systematically in this study, like temperature, stirring time, and their interaction effects, will also affect the average diameter of the particles, as mentioned previously elsewhere^28^.

The interaction plots in Figs 1C and 2C clearly show that the average hydrodynamic diameter of the particles is not dependent on a single factor but rather on a combination of influences of different single factors and their interactions. Fig. 1C shows that there is a strong influence of chitosan concentration on the average hydrodynamic diameter of the particle at a molar ratio of 1.5, but this effect is stronger when a high DA chitosan (codified value 1) is involved. Interestingly, this phenomenon is inverted at a lower molar ratio (Fig. 2C): at a molar ratio of 1, the influence of chitosan concentration on the average hydrodynamic diameter of the particles is stronger when a low DA chitosan (codified value −1) is used.

The influence of DA was also studied with chitosans of lower DA (10%) and higher DA (60%), but no trend in the average hydrodynamic diameter of the particles formed as a function of chitosan concentration at any of the NH_2_/PO_4_ molar ratios studied was observed, indicating that using a chitosan with very low or very high DA gives less control over the particle preparation process.

In conclusion, we identified conditions allowing the preparation of chitosan particles with average hydrodynamic diameters ranging from nano- to micrometer by simply changing the chitosan concentration (and, consequently, the TPP concentration, as the chitosan/TPP ratio was kept constant), as long as the DA of the chitosan was in the range of 20–50%. To our knowledge, the ability to control the diameter of chitosan particles form the nano- to the microscale using ionic gelation has not been reported before. In previous studies, only sub-micrometer particles were obtained^1,7,17^, as beyond a chitosan concentration of 1.5 mg/mL, aggregates were being formed. To verify this simple influence of chitosan concentration on the average hydrodynamic diameter of the particles formed, we repeated the experiments using the conditions identified in the experimental design study but using a larger number of different chitosan concentrations. We used chitosans of DA 20% and 50% at a NH_2_/PO_4_ molar ratio of 1.5, and chitosan of DA 35% at a NH_2_/PO_4_ molar ratio of 1.

**Figure 3:**
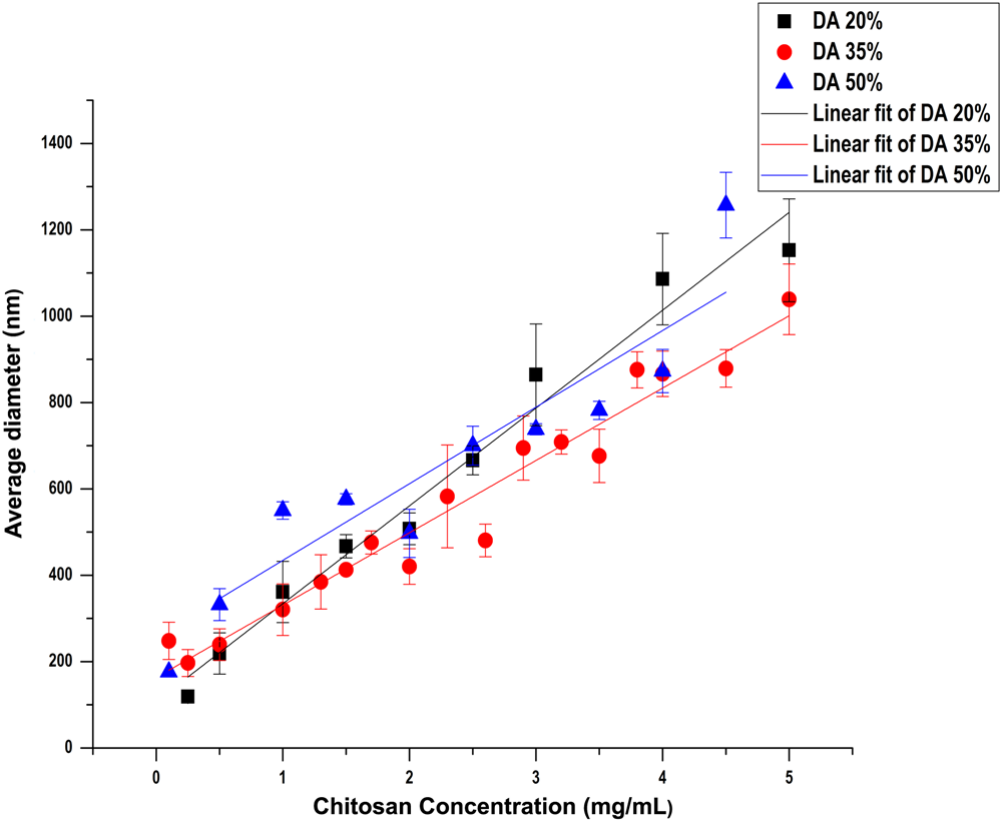
Influence of chitosan concentration on the average hydrodynamic diameter of particles prepared using DA 20% (■), DA 35% (), and DA 50% () with a NH_2_/PO_4_ ratio of 1.5 for DA 20% and 50%, and with a NH_2_/PO_4_ ratio of 1 for DA 35%. The values of R^2^ were 0.97, 0.93, and 0.82 for DA 20%, 35%, and 50%, respectively.

**Figure 4:**
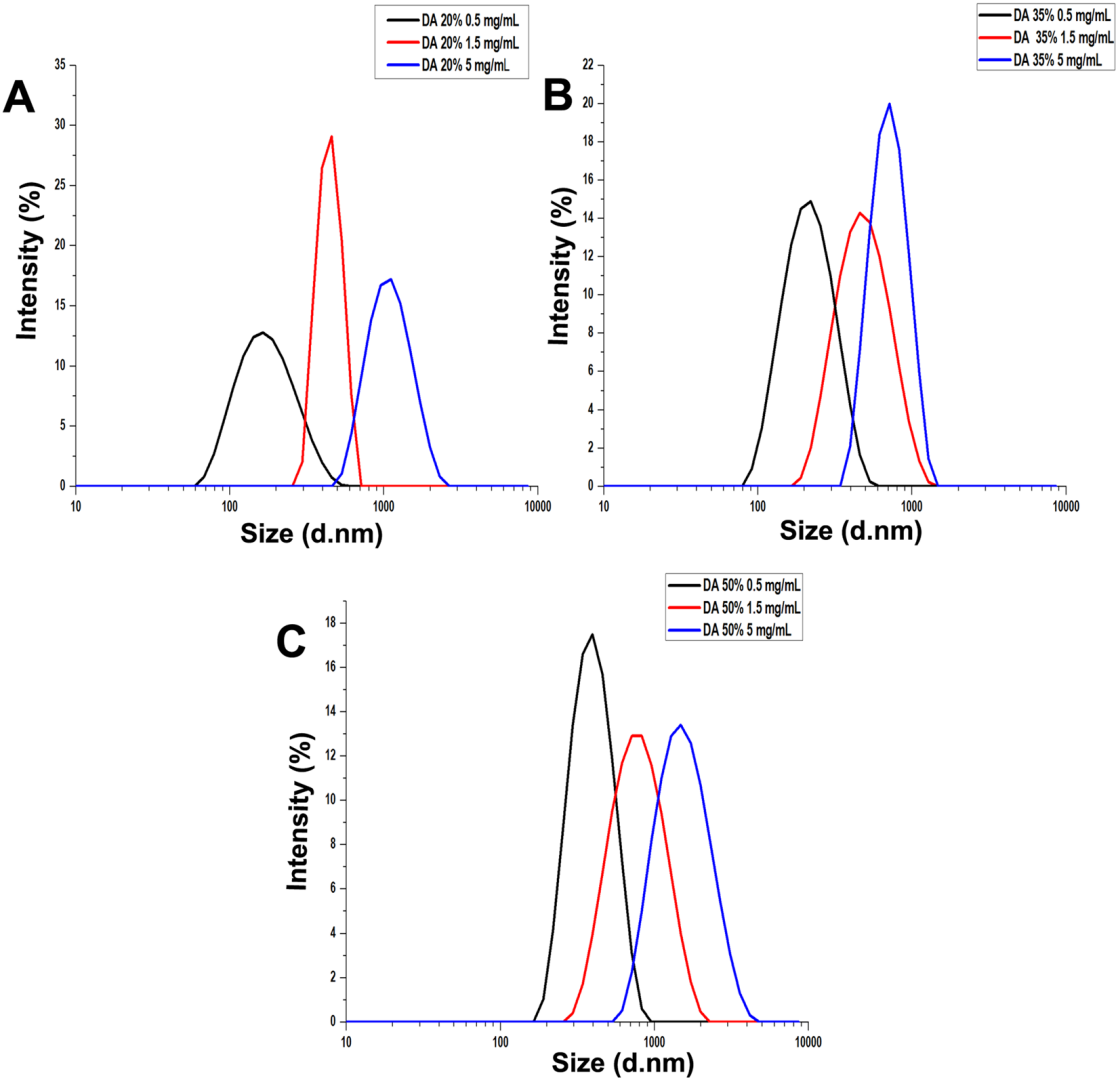
Size distribution by intensity of chitosan-TPP particles prepared from chitosan samples at varying DA and concentration (black 0.5 mg/ mL, red 1.5 mg/mL, blue 5 mg/mL): (**A**) DA 20% with a NH_2_/PO_4_ ratio of 1.5; (**B**) DA 35% with a NH_2_/PO_4_ ratio of 1; (**C**) DA 50% with a NH_2_/PO_4_ ratio of 1.5.

As seen in Fig. 3, the average hydrodynamic diameter of the particles increased with increasing chitosan concentrations in all cases, confirming our earlier conclusion. The average hydrodynamic diameter of the particles ranged from ca. 100 to ca. 1200 nm, with low polydispersity of 0.1–0.4 in all samples (see Supplementary Tables 1–3). Clearly, however, the slope and intercept values observed for the different chitosans differed, indicating that the DA of the chitosan used has an effect on the relationship between chitosan concentration and particle size. Fig. 4 shows the size distribution of particles prepared from low (0.5 mg/mL), medium (1.5 mg/mL), and high (5 mg/mL) chitosan concentrations; the respective cumulant fits are given in Supplementary Fig. S1. It is clearly seen that the size distribution is strongly influenced by both the DA and the concentration of the chitosan under study.

**Figure 5:**
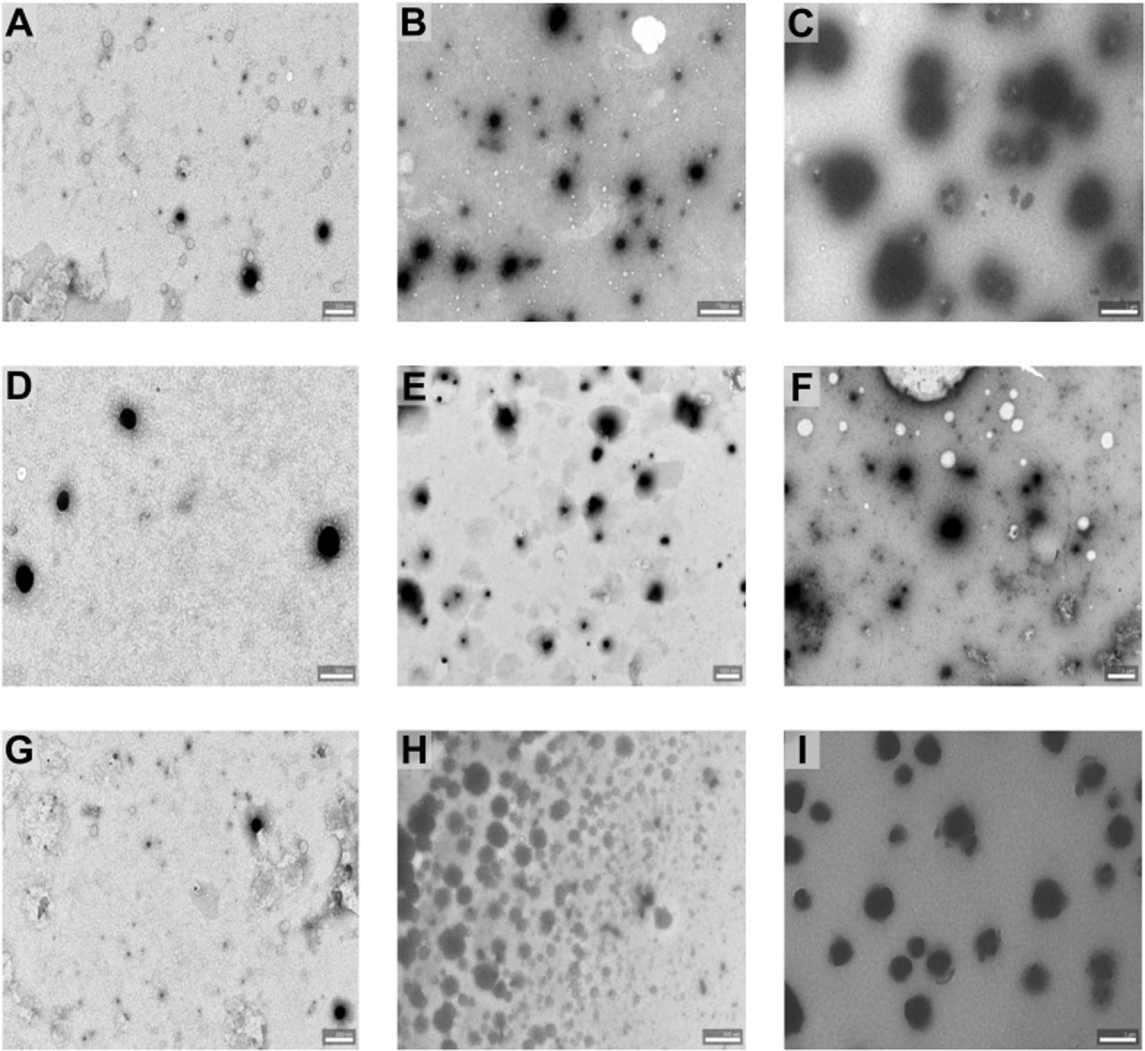
TEM micrographs of chitosan-TPP particles prepared from chitosan samples at varying DA and concentration with a NH_2_/PO_4_ molar ratio of 1.5 for DA 20% and 50%, and with a NH_2_/PO_4_ molar ratio of 1 for DA 35%: (**A**) DA 20% at 0.5 mg/mL chitosan concentration; (**B**) DA 20% at 2.5 mg/mL chitosan concentration; (**C**) DA 20% at 5 mg/mL chitosan concentration; (**D**) DA 35% at 0.5 mg/mL chitosan concentration; (**E**) DA 35% at 2.5 mg/mL chitosan concentration; (**F**) DA 35% at 5 mg/mL chitosan concentration; (**G**) DA 50% at 0.5 mg/mL chitosan concentration; (**H**) DA 50% at 2.5 mg/mL chitosan concentration; (**I**) DA 50% at 5 mg/mL chitosan concentration. Scale bars (**A**,**D**,**G**) = 200 nm; (**B**,**E**,**H**) = 500 nm; (**C**,**F**,**I**) = 1 µm.

Transmission electron micrographs were taken to confirm the spherical morphology of particles prepared at different chitosan concentrations. Figure 5 shows representative TEM micrographs of particles produced at 0.5 mg/mL and 5 mg/mL concentration of chitosan at the optimum NH_2_/PO_4_ molar ratio defined for the respective chitosan. The results confirm our data from DLS, namely the increase in hydrodynamic diameter of the particles with increasing chitosan concentration, and show that the spherical morphology of the particles is not influenced by increasing the chitosan concentration.

The influence of chitosan concentration on the average hydrodynamic diameter of the particles can be related to volume occupied by the polymer coil in a solution. The volume occupied by a polymer in solution can be given as the product of its concentration and its intrinsic viscosity (C[η]), the so-called “coil overlap concentration” which reflects the degree of space occupancy of the polymer coils. In turn, the specific viscosity (η_sp_) is known to scale with the degree of space occupancy. In general, three regimes of concentration-dependence in a double logarithmic plot of η_sp_ *versus* C[η] have been observed in a wealth of polysaccharide random coil systems, generating a “master curve”^29–31^. In the dilute regime, the polymer coils are free to move and do not touch each other but only generate frictional forces. As the concentration increases, they start to touch each other (at C[η] ~1); at even greater concentrations and after some contraction, they finally enter the entanglement regime. The transition from the dilute to the entangled regime is characterized by a change in the slope of the curve and often occurs at coil overlap C[η] ~4. Once in the entangled regime, it is not possible to produce particles.

**Figure 6:**
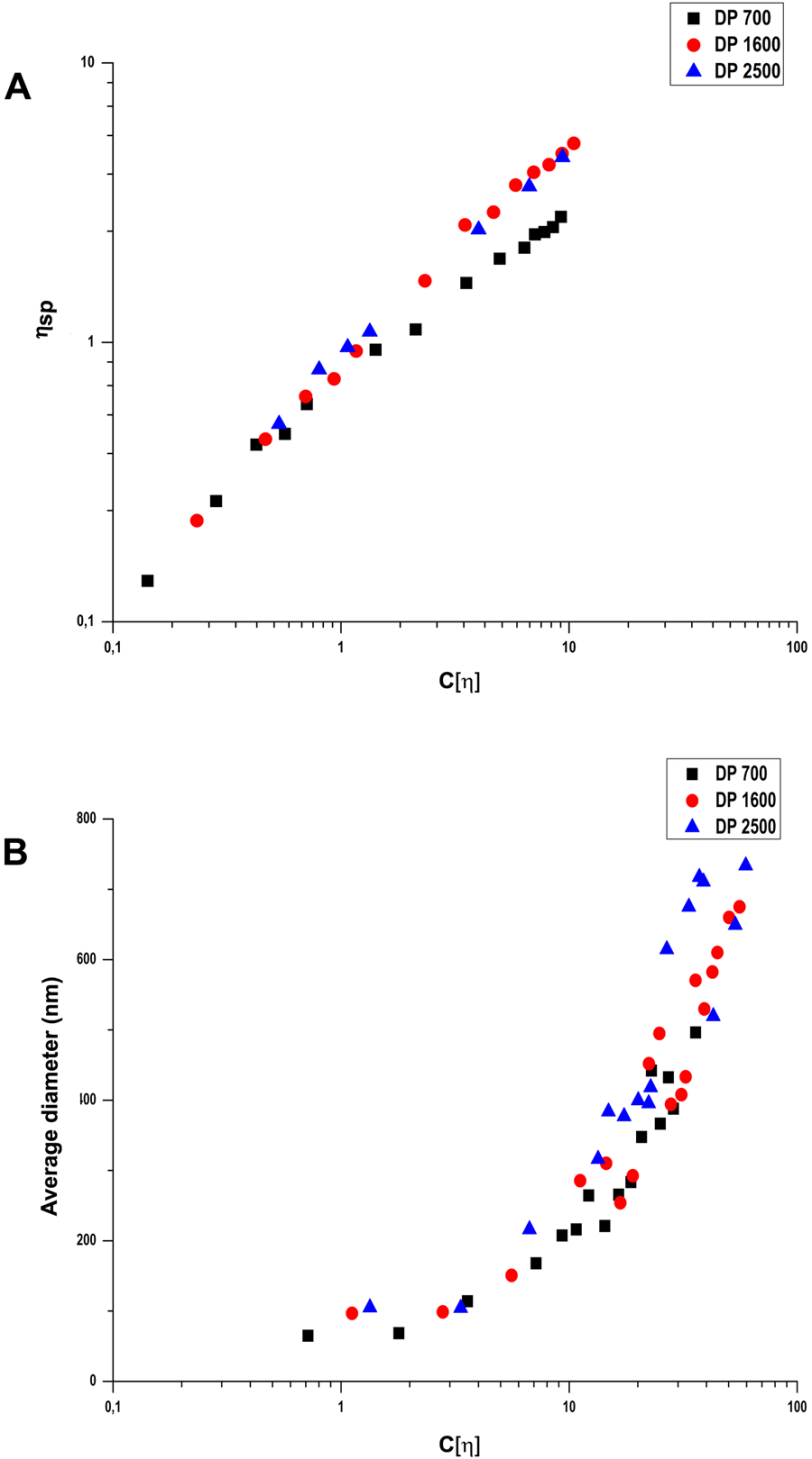
Viscosimetry studies performed on chitosans with similar DA (35%) but varying DP (700, 1600, 2500). (**A**) log-log plot of specific viscosity vs chitosan intrinsic viscosity (**B)** semi-log plot of chitosan intrinsic viscosity vs average hydrodynamic diameter of particles. [η] = intrinsic viscosity, C [η] = concentration times the intrinsic viscosity, η_sp_ = specific viscosity.

With this knowledge on the behavior of polysaccharide systems in solution, we performed viscosimetry studies using chitosans of DA 35% with three different sizes, namely DP 700, 1600, and 2500. Previously, we have confirmed that chitosans of varying DA and DP in dilute aqueous acidic solutions and in the presence of 85 mM NaCl conform to a single “master curve” which experiences a change in slope after the incipient coil contact point (C[η] ~1.27)^24^. When we used this same approach to analyze our data presented here for chitosans of varying DP in dilute acetic acid and in the absence of added salt, we observed that the expected concentration dependence (Fig. 6A) was very different from that of the “master curve” previously described. In contrast to the expected increase in slope after the close contact point (C[η] ~1), the curves for the different chitosans did not exhibit an increase in slope even up to C[η] ~10 (Fig. 6A). Regression analysis including all the different DP values showed that they fall within the confidence interval (see Supplementary Fig. S2). The maximum concentration in these experiments was restricted due to technical difficulties of working at higher concentrations using the rolling ball viscosimeter.

Another striking observation was that the η_sp_ values for the three samples were much lower than those registered before in the presence of electrolyte at equivalent values of C[η]^24^. In a semi-log plot of C[η] versus average hydrodynamic diameter of the particles (Fig. 6B), we observed that particles were formed at degrees of space occupancy varying within a range of two orders of magnitude, namely C[η] ~0.7 to ~70 (equivalent to concentrations between 0.1 and 5 mg/mL). Here we calculated the C[η] values by multiplying the intrinsic viscosity measured for various DP with the concentration used for particle preparation. Notice that there was a clear non-linear dependence between the average hydrodynamic diameter of the particle and the C[η], and within experimental error, the data seemed to conform to a single “master plot”. Also, this dependence seemed to be characterized by two regimes of behavior, namely one between C[η] ~0.7 and ~10, and another one between C[η] ~10 and ~70, with a “breakpoint” centered at C[η] ~10 (Fig. 6B). As mentioned before, previous studies in our group had already described the influence of the intrinsic viscosity on the average diameter of chitosan particles but up to now, these studies were always carried out in the presence of added salt to the solvent (e.g., 85 mM NaCl). Under such conditions, we were not able to obtain particles larger than 500 nm and particle formation occurred only in the dilute and semi-dilute regime (C[η] < ~4).

A possible explanation for the *sui generis* behavior observed for chitosan solutions in the absence of added salt may reside in the self-charge screening effect that occurs as the polymer concentration increases. This self-charge screening effect leads to the gradual contraction of the coils as the concentration increases. The overall consequence of this is that the chitosan remains in the dilute/semi-dilute regime, below the onset of entanglement, over a wide range of concentration (i.e., also degree of coil overlap), even as high as 5 mg/mL (C[η] 70).

To support this hypothesis, particle formation was studied in the presence of NaCl (see Supplementary Fig. S3) at varying concentrations, namely 50 mM, 100 mM, and 150 mM. These conditions were chosen based on a large number of studies describing the use of NaCl to gain greater control on particle preparation and to mimic physiological salinity (100–200 mM). As expected, no particles were formed at the highest concentration of 5 mg/mL in the presence of NaCl. Also, the average hydrodynamic diameter of the particles at lower concentrations decreased drastically in the presence of salt, mainly due to reduced stiffness and charge repulsion of chitosan chains in the presence of monovalent salts leading to the formation of more compact particles, in accordance with previous studies which had also shown that multivalent ions lead to the formation of even more highly compact polyelectrolyte structures^6,16,23,33^. The effect of increasing ionic strength on the contraction of polyelectrolyte chains in dilute solution is known to be more pronounced, the more flexible the polyelectrolyte chain^34^.

## Conclusion

In the present study, we examined the influence of various factors - namely the chitosans DA and DP, polymer concentration, degree of space occupancy, and degree of crosslinking with TPP (NH_2_/PO_4_ molar ratio) - on the ability of chitosans to form particles by ionic crosslinking and the influence of such parameters on the average hydrodynamic diameter of the particles formed. It was seen that for every chitosan, a specific NH_2_/PO_4_ molar ratio was required to obtain particles ranging from nano- to micrometer in diameter. An unprecedented finding in our study is that chitosan can remain in the dilute regime even at concentrations as high as 5 mg/mL, thus driving the formation of large particles, which cannot be obtained in the presence of salts such as NaCl.

## Electronic supplementary material

Supplementary Information
