# Supplementary material for: Parameters influencing the size of chitosan-TPP nano- and microparticles
Source: Sci Rep. 2018 Mar 16;8:4695. doi: 10.1038/s41598-018-23064-4 (PMC5856823; doi:10.1038/s41598-018-23064-4)
Supplement: Supplementary file 1 — Supplementary Information [file 41598_2018_23064_MOESM1_ESM.pdf]

Supplementary

Parameters influencing the size of chitosan-TPP nano- and microparticles

Sruthi Sreekumar, Francisco M. Goycoolea[++], Bruno M. Moerschbacher*, Gustavo R. Rivera-Rodriguez

Institute for Biology and Biotechnology of Plants – IBBP,

University of Münster – WWU, Schlossplatz 8, 48143 Münster, Germany

[++]*Current affiliation: School of Food Science and Nutrition. University of Leeds, LS2 9JT, U.K.*

**To whom correspondence should be addressed at moersch@uni-muenster.de*

Supplementary Table S1. Summary of the average size, size distribution (PDI), and PDI width of chitosan particles prepared using a chitosan of DA 20% with NH_2/PO_4 ratio of 1.5 at varying concentrations. Data shown are means ± SD from three independent experiments.

chitosan concentration (mg/mL)	average size (nm)	PDI	PDI width (nm)
0.25	119±6	0.13±0.014	44±0,6
0.5	219±48	0.27±0.00	115±35
1	361±71	0.17±0.10	134±43
1.5	467±27	0.19±0.10	192±81
2	508±37	0.13±0.00	181±12
2.5	666±34	0.23±0.00	326±17
3	864±118	0.26±0.03	438±38
4	1086±106	0.29±0.02	584±73
5	1153±119	0.28±0.03	607±30

Supplementary Table S2. Summary of the average size, size distribution (PDI), and PDI width of chitosan particles prepared using a chitosan of DA 35% with NH_2/PO_4 ratio of 1 at varying concentrations. Data shown are means ± SD from three independent experiments.

chitosan concentration (mg/mL)	average size (nm)	PDI	PDI width (nm)
0.25	197±31	0.25±0.01	100±18
0.5	239±37	0.26±0.03	123±22
1	321±60	0.29±0.02	174±38
1.3	385±63	0.28±0.01	196±46
1.5	413±3	0.26±0.08	215±63
1.7	476±27	0.22±0.02	225±23
2	420±41	0.25±0.05	210±10
2.3	583±119	0.20±0.04	240±77
2.6	481±38	0.29±0.04	262±41
2.9	695±74	0.29±0.10	378±107
3.5	677±62	0.08±0.06	238±14
5	863±40	0.54±0.12	632±62

Supplementary Table S3. Summary of the average size, size distribution (PDI), and PDI width of chitosan particles prepared using a chitosan of DA 50% with NH_2/PO_4 ratio of 1.5 at varying concentrations. Data shown are means ± SD from three independent experiments.

chitosan concentration (mg/mL)	average size (nm)	PDI	PDI width (nm)
0.5	332±37	0.28±0.04	178±21
1	550±20	0.34±0.05	322±39
1.5	576±13	0.29±0.05	310±7
2	497±56	0.26±0.01	258±37
2.5	700±45	0.28±0.04	373±26
3	738±13	0.29±0.01	402±18
3.5	782±21	0.28±0.01	438±32
4	873±50	0.34±0.07	508±36
4.5	1257±76	0.40±0.07	665±26

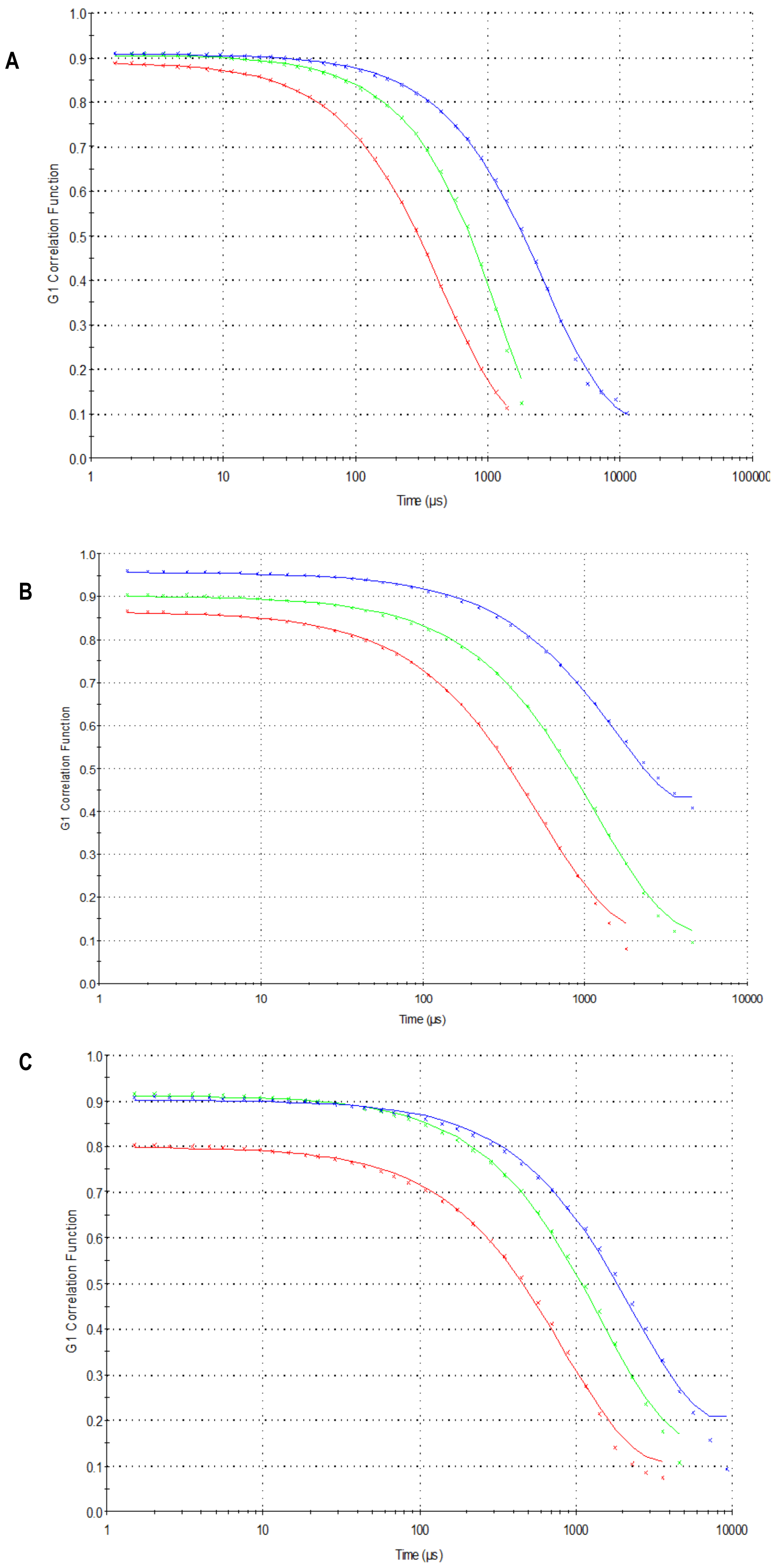

Supplementary Figure S1. Representative cumulant analysis graphs of chitosan-TPP particles prepared from chitosan samples at varying DA and concentration (red 0.5 mg/mL, green 1.5 mg/mL, blue 5 mg/mL) as: A) DA 20% with a NH_2/PO_4 ratio of 1.5; **B)** DA 35% with a NH_2/PO_4 ratio of 1; **C)** DA 50% with a NH_2/PO_4 ratio of 1.5.

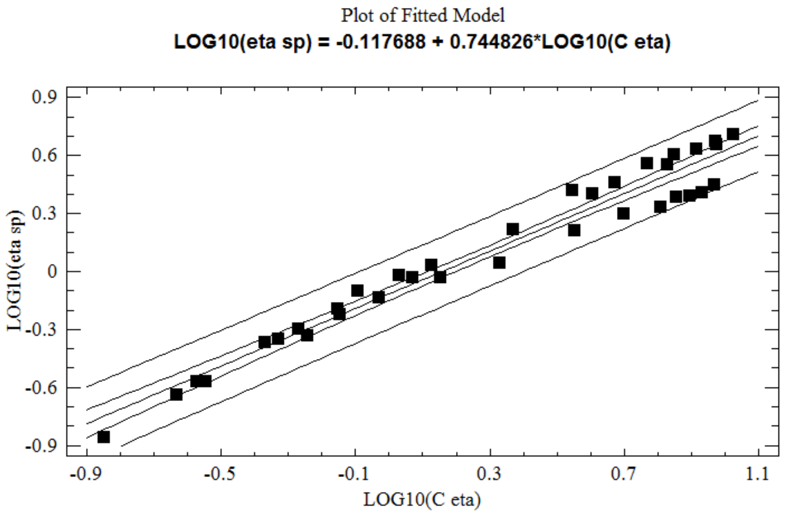

Supplementary Figure S2. Regression analysis of the influence of C[η] over η_{sp} including all the values obtained for DP 700, 1600, and 2500. Shown here is the regression graph including the 95% confidence interval.

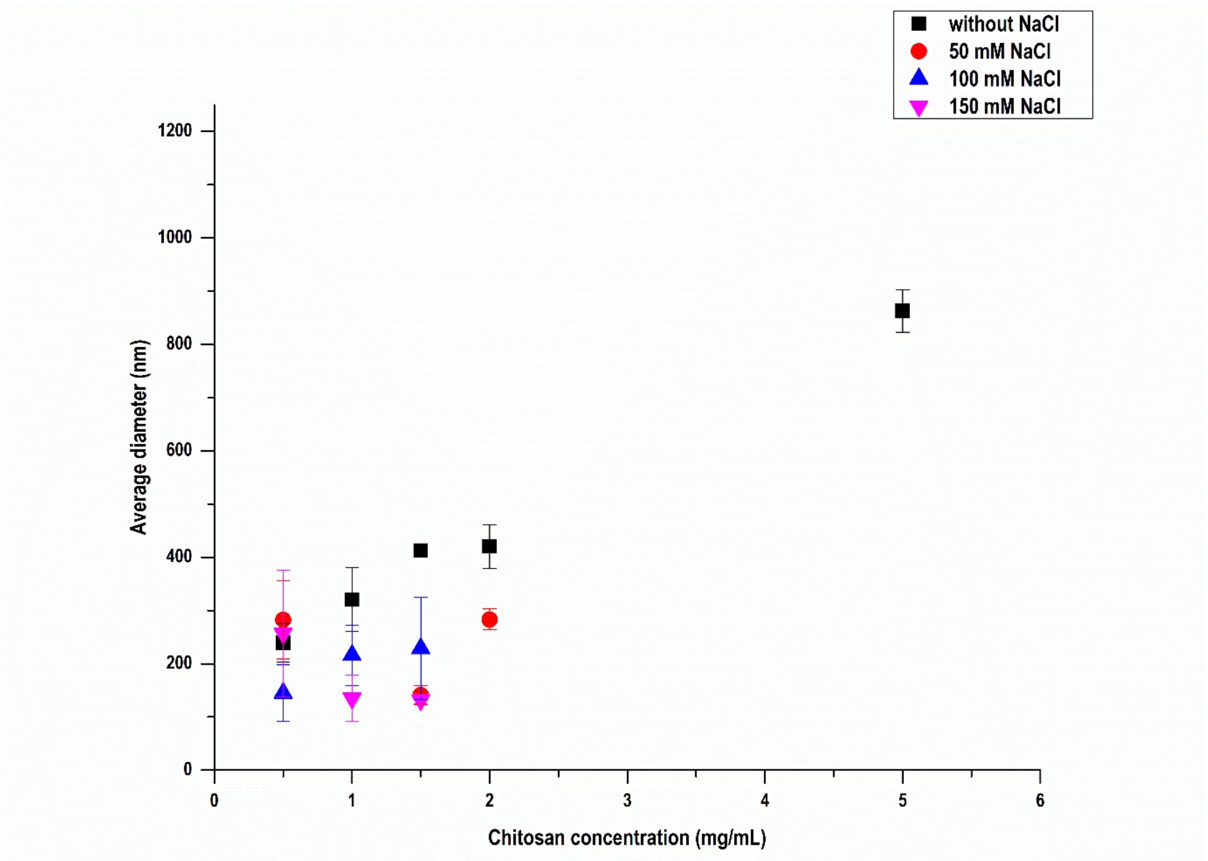

Supplementary Figure S3. Influence of varying salt concentrations (without added salts (■); or with 50 mM (●); 100 mM (▲), and 150 mM (▼) NaCl) on the average diameter of chitosan particles. All particles were prepared at NH_2/PO_4 ratio of 1 using chitosan DA 35% with concentrations ranging from 0.5 mg/mL to 5 mg/mL.
